# Heme Oxygenase Isoforms Differ in Their Subcellular Trafficking during Hypoxia and Are Differentially Modulated by Cytochrome P450 Reductase

**DOI:** 10.1371/journal.pone.0035483

**Published:** 2012-04-24

**Authors:** Monika Linnenbaum, Mareike Busker, Jan R. Kraehling, Soenke Behrends

**Affiliations:** Department of Pharmacology, Toxicology and Clinical Pharmacy, University of Braunschweig–Institute of Technology, Braunschweig, Germany; Universidade Federal do Rio de Janeiro, Brazil

## Abstract

Heme oxygenase (HO) degrades heme in concert with NADPH cytochrome P450 reductase (CPR) which donates electrons to the reaction. Earlier studies reveal the importance of the hydrophobic carboxy-terminus of HO-1 for anchorage to the endoplasmic reticulum (ER) which facilitates the interaction with CPR. In addition, HO-1 has been shown to undergo regulated intramembrane proteolysis of the carboxy-terminus during hypoxia and subsequent translocation to the nucleus. Translocated nuclear HO-1 was demonstrated to alter binding of transcription factors and to alter gene expression. Little is known about the homologous membrane anchor of the HO-2 isoform. The current work is the first systematic analysis in a eukaryotic system that demonstrates the crucial role of the membrane anchor of HO-2 for localization at the endoplasmic reticulum, oligomerization and interaction with CPR. We show that although the carboxy-terminal deletion mutant of HO-2 is found in the nucleus, translocation of HO-2 to the nucleus does not occur under conditions of hypoxia. Thus, we demonstrate that proteolytic regulation and nuclear translocation under hypoxic conditions is specific for HO-1. In addition we show for the first time that CPR prevents this translocation and promotes oligomerization of HO-1. Based on these findings, CPR may modulate gene expression via the amount of nuclear HO-1. This is of particular relevance as CPR is a highly polymorphic gene and deficiency syndromes of CPR have been described in humans.

## Introduction

Heme oxygenase (HO) is the only known enzyme degrading pro-oxidant heme to the antioxidant biliverdin, iron and carbon monoxide [1]. This reaction requires three mol of oxygen and seven electrons supplied by NADPH cytochrome P450 reductase (CPR) [2]. There are two relevant isoforms described in the literature: The inducible HO-1 (33 kDa) is the predominant isoform in liver and spleen [3], [4]. The constitutive HO-2 (36 kDa) is mainly found in brain and testis [5]. Both HO-isoforms as well as the CPR are anchored with a stretch of hydrophobic amino acids to the outer membrane of the endoplasmic reticulum [6], [7]. Early experiments with HO-1 obtained from rat liver have shown that this membrane anchor can be cleaved from membranes by a low concentration of trypsin resulting in a 28 kDa form [8]. It was later observed that expression of full length HO-1 in *E. coli* leads to a 32 kDa form in membranes and a carboxy-terminally deleted 30 kDa form in the soluble fraction [9]. More recent data indicate that these anchorless carboxy-terminally deleted soluble HO-1 isoenzymes are formed *in vivo* under conditions of hypoxia [10]. Although crucial amino acids for binding to CPR have been mapped to the central part of HO-1 [11], [12], there is also evidence to suggest that the hydrophobic tail in HO-1 contributes to increased binding affinity for CPR [13], [14]. Thus, regulated intramembrane proteolysis of the carboxy-terminus of HO-1 under conditions of hypoxia will also weaken the functional interaction with CPR leading to a loss in enzyme activity [10].

HO-1 and HO-2 share a high degree of sequence homology: 45% in total and 59% in the highly conserved region (see “Alignment S1”). The carboxy-terminal membrane anchor shows similarities but is less conserved (∼15%). A carboxy-terminally deleted form of HO-1 has been successfully expressed in *E. coli* [15]. Subsequent crystallization has greatly facilitated the elucidation of the catalytic mechanism [16]. The characterization of the heme regulatory motifs in HO-2 has also been based on carboxy-terminal deletion variants [17]. So far expression of HOs with subsequent purification has only been done in *E. coli* bacteria. For the study of full length HOs that are anchored to the endoplasmic reticulum with their carboxy-terminus such a prokaryotic expression system is not ideal. The current study is the first to address expression and purification of both HO isoforms and their carboxy-terminal deletion mutants using a eukaryotic expression system. This includes the study of their subcellular localization under normoxic and hypoxic conditions using fluorescent fusion proteins and their interaction with CPR by fluorescence resonance energy transfer (FRET) and co-purification.

## Materials and Methods

### Materials

Unless stated otherwise, chemicals were purchased in highest purity from Sigma-Aldrich (Taufkirchen, Germany). Cell culture media and transfection reagents were received from Invitrogen (Darmstadt, Germany) or PAA (Pasching, Austria). All restriction enzymes were obtained from New England Biolabs (Frankfurt/Main, Germany). pECFP, pEGFP and pEYFP vectors were from Clontech (Heidelberg, Germany). The pFastBac™1 and pCR® 2.1 TOPO® vectors were from Invitrogen. The oligonucleotides were from Biomers (Ulm, Germany).

### Cloning of HOs, CPR and biliverdin reductase

HOs were cloned from human placenta cDNA (FirstChoice® PCR ready human placenta cDNA, Ambion, Austin, USA) using the following primers: HO-1 sense 5′-cccagcaccggccggatggag-3′ and HO-1 antisense 5′-ttcagtgcccacggtaaggaagc-3′; HO-2 sense 5′-cagaggagcgagacgagcaag-3′and HO-2 antisense 5′-aggggtaggccagtggtcagtcg-3′. The PCR products were cloned into the pCR® 2.1 TOPO® vector before the corresponding inserts were transferred in the pFastBac™1 vector. The respective baculoviruses were constructed accordingly to the BAC-TO-BAC™ system (Invitrogen). Human CPR cDNA in pUV I was kindly provided by Dr. F. Gonzalez (National Cancer Institute, National Institutes of Health, Rockville, USA). A baculovirus encoding CPR was a generous gift of Dr. D. Schwarz (Berlin, Germany). Human biliverdin reductase (hBVR) was cloned as previously described [18]. For the carboxy-terminally deleted HO-1 and HO-2 isoforms a stop codon was inserted after amino acids 265 or 288, respectively using the QuickChange® Lightning Site-Directed Mutagenesis Kit (Agilent Technologies, Waldbronn, Germany) with specific primers. The sense primers were synthesized as follows: 5′-cacccgctcccaggcttagcttctccgatgg-3′ (HO-1) and 5′-ccttccgaacagctatgtgagtgctgaggaagcccag-3′ (HO-2). For purification assays a *Strep*-tag II (IBA BioTAGnology, Göttingen, Germany) was fused to the amino-terminus of all HO-variants in pFastBac™1 (S-HOs) by molecular cloning. HOs were fluorescently labeled with CFP (cyan fluorescent protein), YFP (yellow fluorescent protein) or GFP (green fluorescent protein) at their amino-terminus (FP-HOs) using the corresponding vectors pECFP-C1, pEYFP-C1 and pEGFP-C1, while CPR was labeled with CFP and YFP at its carboxy-terminus (CPR-FP) using pECFP-N1 and pEYFP-N1.

### Cell culture

HEK293 cells were cultivated in Dulbecco's Modified Eagle Medium-High Glucose with 10% fetal bovine serum and 1% penicillin/streptomycin at 37°C with 5% CO_2_. For microscopy assays cells were seeded in imaging plates with a special glass bottom (zell-kontakt, Nörten-Hardenberg, Germany). Lipofectamine LTX was used for transfection with cDNA encoding for fluorescence protein labeled HO and CPR. The incubation time was at least 48 h.

For the cultivation of Sf9 cells Sf-900 II serum-free medium with 10% fetal bovine serum and 1% penicillin/streptomycin was used. Cells were grown in suspension at 27°C with 140 rpm on a rotating incubator (New Brunswick scientific, Edison, USA). The cells were harvested after 72 h of infection with respective recombinant baculoviruses.

### Purification and gel filtration of HOs

Cell pellets from 500 ml Sf9 cell suspension (2×10^6^ cells/ml) infected with recombinant baculoviruses were resuspended in 15 ml TEA (triethanolamine)-lysis buffer (50 mM) containing 1 mM EDTA and cOmplete™ EDTA-free protease inhibitor cocktail tablets (Roche, Mannheim, Germany). After sonication the homogenate was incubated with avidin (2 nM, IBA BioTAGnology) for 30 min at 4°C. To obtain the cytosolic fraction the samples were centrifuged at 15,000 g for 2 h at 4°C and filtered with a 0.4 µm sterile filter (Sarstedt, Nümbrecht, Germany). A chromatography system from GE Healthcare Life Sciences (ÄKTA™purifier, Freiburg, Germany) was used with a *Strep*-Tactin® Superflow® high capacity column (2-ml volume, IBA BioTAGnology). The following buffers were used: *Washing Buffer:* 100 mM Tris, 500 mM NaCl, 1 mM EDTA, 1 mM benzamidine-HCl, pH 8.0; *Elution Buffer*: 100 mM Tris, 500 mM NaCl, 1 mM EDTA, 1 mM benzamidine-HCl, 2.5 mM D-desthiobiotin, pH 8.0 and *Regeneration Buffer*: 100 mM Tris, 150 mM NaCl, 1 mM EDTA, 1 mM HABA, pH 8.0. The fractions showing absorption at 280 nm were collected and concentrated with Amicon® ultra centrifugal filter devices (10 kDa) (Millipore, Billerica, USA). The protein concentration was measured with a nanophotometer (Implen, Munich, Germany) against elution buffer blank.

For gel filtration 150 µg purified protein were used. Gel filtration buffer contained 50 mM TEA-HCl and 250 mM NaCl, pH 8.0. The gel filtration was carried out using the ÄKTA™purifier with the gel filtration column Superdex 200 10/300 GL (GE Healthcare Life Sciences). For calibration the gel filtration LMW calibration kit (GE Healthcare Life Sciences) was used. The void volume was determined using Blue Dextran (2000 kDa). For size determination the standard linear equation was calculated based on the calibration of the column.

### Heme oxygenase activity assay

Harvested Sf9 cells were resuspended in 50 mM TEA-HCl, pH 7.6, containing cOmplete™ EDTA-free protease inhibitor cocktail tablets (Roche). For cell lysis the cell suspension was sonicated. The resulting homogenate was either used directly or a cytosolic fraction was obtained by centrifugation at 21,000 g for 30 min at 4°C. For the activity assay 200 µl of homogenate or cytosolic fraction was used. Purified enzyme was assayed using a total protein amount of 25 µg. The HO-assay mixture contained phosphate buffered saline-magnesia buffer, 1 mM glucose-6-phosphate, 0.75 units/ml glucose-6-phosphate dehydrogenase, 1 mM NADPH (Applichem, Darmstadt, Germany), 25 µM freshly dissolved hemin (hemin stock solution dissolved in 2 N NaOH diluted 1∶200 with phosphate buffered saline-magnesia buffer), recombinantly expressed (exogenous) CPR and human biliverdin reductase (hBVR), which is necessary for the reduction of biliverdin to bilirubin. The final volume was 500 µl. The incubation was carried out at 37°C for 1 h in the dark. The formed bilirubin was extracted with 350 µl chloroform by mixing thoroughly for 30 sec and centrifugation at 10,000 rpm for 5 min at 4°C. This step was repeated before absorbance of the chloroform phase was measured with a Varian spectrophotometer (Agilent Technologies) at 464 nm and 530 nm. To calculate the HO-activity (pmol/h/mg) a molar extinction coefficient of 40 mM/cm was used [19].

### SDS-PAGE, immunodetection and Coomassie staining

Laemmli sample buffer was added to 5 µg of purified protein samples or 80 µg of cytosolic fractions. The proteins were separated on 10% SDS polyacrylamide electrophoresis gels which were used for either Western blotting or Coomassie staining. A prestained marker (Fermentas, St. Leon-Rot, Germany) was used for size control. For immunodetection the separated gels were blotted on nitrocellulose membranes. After reversible staining with Ponceau S the membranes were blocked with 5% non-fat dry milk in TBST (10 mM Tris, 150 mM NaCl, 0,1% (*v/v*) Tween®20). The HO-1 and HO-2 antibodies (Stressgen, Enzo Life Sciences, Lörrach, Germany) and the CPR antibody (Abcam, Cambridge, England) were incubated for 2 h in TBST with 1% non-fat dry milk, washed three times and then incubated with an anti-rabbit IgG horse radish peroxidase linked antibody (Cell Signaling, Darmstadt, Germany). Chemiluminescence was examined using the lumi-light^plus^ western blotting substrate (Roche). For Coomassie staining the gels were incubated overnight with Coomassie Brilliant Blue solution (0.02% Coomassie Brilliant Blue -G250, 5% aluminium sulfate, 10% ethanol, 2% orthophosphoric acid). Excess staining solution was removed with washing solution (10% ethanol, 2% orthophosphoric acid) for more than 1 h [20]. Detection was carried out using a colour scanner.

### FRET measurements using the sensitized emission method

A Varian spectrofluorometer (Agilent Technologies) was used for FRET studies. Homogenates and cytosolic fractions from Sf9 cells infected with respective baculoviruses (CFP-HO-1; CFP-HO-1ΔC266; CFP-HO-2; CFP-HO-2ΔC289 and CPR-YFP) were tested at 37°C in a heated cuvette holder. FRET measurements were based on the sensitized emission method using three channels. In the donor channel an excitation wavelength of 436 nm and an emission wavelength of 476 nm were selected. For detection of YFP the acceptor channel with an excitation wavelength of 515 nm and an emission wavelength of 527 nm was used. This resulted in a FRET channel with an excitation wavelength of 436 nm and an emission wavelength of 527 nm. The excitation- and emission-slits were 5 nm.

The samples were diluted with lysis buffer to similar fluorescence intensity in the YFP-channel before starting measurements in all three channels. To make up for unspecific fluorescence of cytosolic components the background was determined with uninfected Sf9 cytosol. The background was proportional to the protein concentration of the samples and was substracted from the measured intensities. The calculated intensities were designated in the following equations as *CFP* and *YFP*. The corrected FRET (FRET^c^) considered bleed-through factors for CFP and YFP and was calculated using the equation: FRET^c^ = FRET−(0.446×*CFP*)−(0.0177×*YFP*) [21]. The bleed-through factors were determined by the ratio of FRET channel intensity to corresponding CFP or YFP channel intensities. The factors corresponded to those described in literature [22]. To determine the degree of interaction the FRET efficiency (E) was calculated according to the equation: E = 1−[CFP/(CFP+FRET^c^×Q_d_/Q_a_)] with the quantum yields for the donor CFP (Q_d_ = 0.4) and the acceptor YFP (Q_a_ = 0.61) [23], [24].

### In vivo fluorescence lifetime imaging (FLIM)

HEK293 cells were analyzed at 37°C on the confocal microscope system A1 from Nikon (Nikon Europe, Kingston, England) equipped with a Ti-E microscope and an incubation chamber (Okolab, Naples, Italy) using a 40× oil immersion objective (NA 1.4, Nikon). Fluorescence lifetimes were measured in cells expressing only the FRET donor, and in cells expressing the combination of FRET donor and acceptor. Cells were excited using a 405 nm pulsed laser. FLIM images of fluorescent cells were recorded with a four channel time gated detection system (LiMo module, Nikon) using a CFP bandpass filter (475/20 nm). The intensities were fitted using a mono-exponential function. The FRET efficiency (E) was calculated according to the equation: E = 1−(τ_DA_/τ_D_), where τ_DA_ is the mean fluorescence lifetime of cells co-expressing FRET donor and acceptor and τ_D_ is the mean fluorescence lifetime of cells expressing FRET donor only [24].

### Detection of translocation using a confocal laser scanning microscope

Transfected HEK293 cells were analyzed with the same microscope used for FLIM. The measurements were made at 37°C with a 60× oil immersion objective using the 488 nm laser for GFP and a 525/50 bandpass filter (emission range of 500–550 nm). CFP and YFP were excited with 457 nm and 514 nm, respectively; the corresponding emissions were measured between 464–499 nm and 525–555 nm using the bandpass filters 482/35 and 540/30.

Cells were incubated under hypoxic conditions for 42 h using an O_2_/CO_2_ incubator (Sanyo, Munich, Germany) with 1% O_2_, 5% CO_2_ and 94% N_2_ at 37°C.

### Statistical analysis

The results are expressed as means ± SEM of three independent experiments. Statistical significance was calculated using the Student's t-test with p<0.05.

## Results

### Differences in CPR dependent HO isoenzyme activity

**Figure 1:**
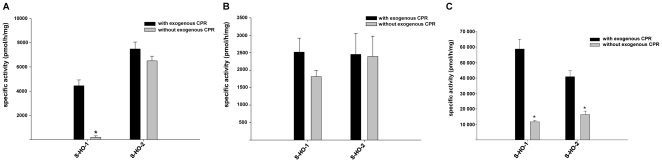
Comparison of enzyme activity of full-length HO isoforms. Measurements were made in the presence (black columns) and absence (grey columns) of exogenous CPR. (A) HO activity measured in cytosolic fractions from Sf9 cells expressing the indicated HO variants. (B) Same experiments made in homogenates from Sf9 cells. (C) Enzyme activity assay after purification using the *Strep*-tag/Streptavidin system. Data are shown as means ± SEM (n = 3, *p<0.05).

HO-1 and HO-2 were expressed in Sf9 cells. Measurement of HO activity in the absence and presence of exogenous CPR in cytosolic fractions showed a significant difference between both isoforms: While HO-1 activity was dependent on addition of exogenous CPR, HO-2 was equally active without addition of exogenous CPR (Figure 1A). When homogenates of Sf9 cells were used instead of cytosolic fractions both HO-1 and HO-2 were catalytically active regardless of exogenous CPR (Figure 1B). This indicates that endogenous CPR activity in Sf9 cells can support HO activity, but the functional interaction is lost for HO-1 upon the preparation of cytosol. After purification both HO isoforms needed exogenous CPR for full activity (Figure 1C).

**Figure 2:**
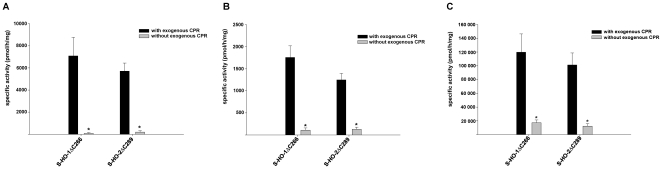
Comparison of enzyme activity of the carboxy-terminally deleted HO isoforms. Measurements were made in the presence (black columns) and absence (grey columns) of exogenous CPR. (A) Activity measured in the cytosolic fractions from Sf9 cells expressing the HO mutants. (B) Measurement in homogenates from infected Sf9 cells. (C) Enzyme activity assay after purification using the *Strep*-tag/Streptavidin system. Data are shown as means ± SEM (n = 3, *p<0.05).

We hypothesized that the different ability of HO-1 and HO-2 to use CPR activity occurring endogenously in Sf9 cell cytosol, may be due to a loss of the membrane anchor in HO-1 upon preparation of cytosolic fractions. If this was true carboxy-terminal deletion mutants of HO-1 and HO-2 should all lose their ability to use endogenous Sf9 cell CPR activity and behave similarly in response to exogenous CPR. Figure 2 shows that this is the case not only in cytosol (Figure 2A), but also in homogenate (Figure 2B) and after purification (Figure 2C). This supports the idea that the membrane anchor is crucial for functional interaction with endogenous CPR activity [13], [14] and makes it likely that HO-1 in Sf9 cytosol has lost part of the membrane anchor similar to what has been described in the literature [9].

### Co-purification of HO variants with CPR

**Figure 3:**
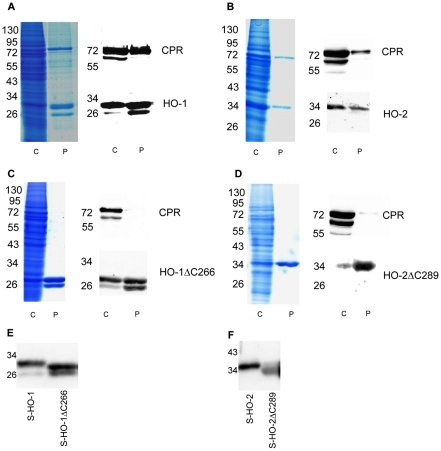
Coomassie staining and immunoblot analysis of co-infections of HO-isoforms and CPR in Sf9 cells. (A) S-HO-1/CPR coinfection (B) S-HO-2/CPR coinfection (C) S-HO-1ΔC266/CPR coinfection (D) S-HO-2ΔC289/CPR coinfection. The respective lanes C show cytosolic fractions (80 µg protein) of the co-infections and lanes P samples after purification (5 µg). For purification *Strep*-tagged HO isoforms were used in combination with untagged CPR. Coomassie staining was used to control the degree of purification. Western blot analysis was performed using specific antibodies against CPR (upper blot) and the HO-isoforms 1 and 2 (lower blot). Panel E and F show a HO-Western-blot analysis for direct comparison of full-length and truncated isoforms. The molecular weight of the proteins was specified in kDa.

In order to compare CPR binding to HO-isoforms with and without the carboxy-terminal membrane anchor, we tested whether the interaction between HO and CPR is strong enough to allow co-purification. A *Strep*-tag was attached to the amino-terminus of the HO-variants. After co-infection of Sf9 cells with recombinant baculoviruses encoding CPR and the respective *Strep*-tagged HO variants, the proteins were purified to apparent homogeneity by affinity chromatography. The Coomassie stained polyacrylamide gels are shown in Figure 3. In the range of 30 kDa two bands for the HO-1 protein were apparent for full length HO-1 (Figure 3A, left panel). Both of these bands were detectable with an HO-1 antibody directed against several epitopes between amino acids 1 and 261 (Figure 3A, right panel). For the carboxy-terminal deletion mutant of HO-1 a similar double band was apparent (Figure 3C). The upper bands in Figure 3 A and C were different in molecular weight (Figure 3E) and corresponded to the respective translation product of HO-1 and HO-1ΔC266. The lower bands in Figure 3A and C were identical in size (Figure 3E). This indicates that the recombinantly expressed carboxy-terminal deletion variant of HO-1 likely corresponds to the described carboxy-terminally deleted 30 kDa form [9] while the lower bands in Figure 3A and C correspond to the 28 kDa cleavage product described by Yoshida et al. 1991 [8].

For HO-2 a single band was apparent slightly above 34 kDa and at 34 kDa for the carboxy-terminal deletion mutant of HO-2 (Figure 3B and D, left panel). As expected these bands were detected by the respective antibody against HO-2 (Figure 3B and D, right panel). For direct comparison of the molecular weight see Figure 3F). The protein preparations containing the full-length isoforms HO-1 and HO-2 showed an additional band at the height above 72 kDa (Figure 3A and B, left panel). This corresponds to the size of CPR (78 kDa) and was additionally identified as CPR by Western blotting (Figure 3A and B, right panel). In the purified preparations from Sf9 cells co-infected with recombinant baculoviruses encoding CPR and the respective carboxy-terminal deletion mutants of HO, CPR was not detectable by Coomassie stain (see Figure 3C and D, left panel). Extremely faint bands were detectable for CPR in the respective samples by Western blotting (see Figure 3C and D, right panel). This indicates that although the carboxy-terminal deletion mutants of HO retain a residual capacity to interact with CPR, the quantitative physical interaction between HO and CPR is lost without the HO-membrane anchor.

### Enzyme activity of purified HO variants co-expressed with CPR

**Figure 4:**
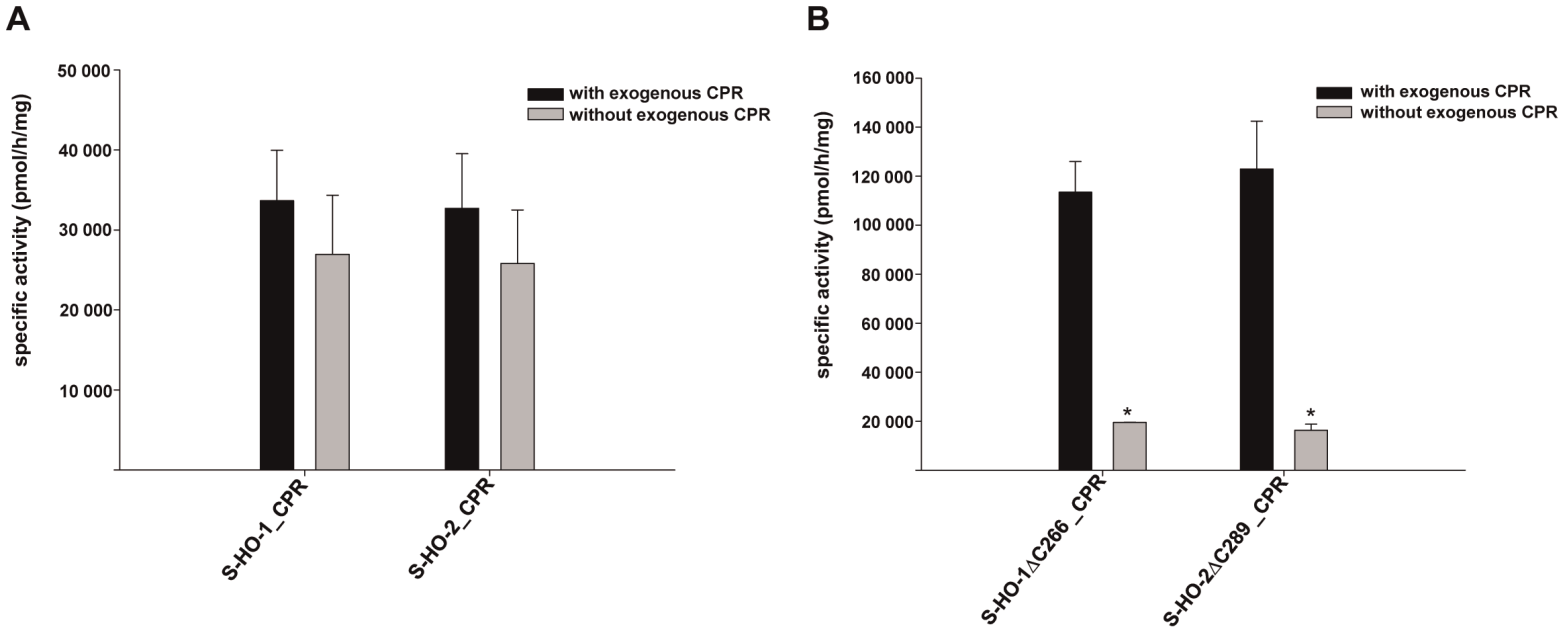
Specific enzyme activity assay of HO-CPR co-infections after purification. Measurements were made in the presence (black columns) and absence (grey columns) of exogenous CPR. (A) Co-purifications of full-length *Strep*-tagged HO-1 and HO-2 (B) Carboxy- terminally deleted HO isoforms. Data are shown as means ± SEM (n = 3, p<0.05).

The difference in quantitative physical interaction between purified full length HO co-expressed with CPR versus purified HO-deletion mutants co-expressed with CPR should also result in a functional difference in the responsiveness of the enzyme activity against exogenous CPR. As expected exogenous CPR added to the purified samples had no significant influence on the catalytic activity of the full length enzymes (Figure 4A), but significantly increased enzyme activity for the carboxy-terminal deletion mutants (Figure 4B). This indicates that the physical interaction of CPR with the full length enzymes (see Figure 3A and B) also results in a functional interaction between HO and CPR. Maximal enzyme activity under optimal conditions was about three times higher for the carboxy-terminal deletion mutants than for the full length enzymes (note different axes in Figure 4).

### Influence of CPR on HO oligomerization

**Figure 5:**
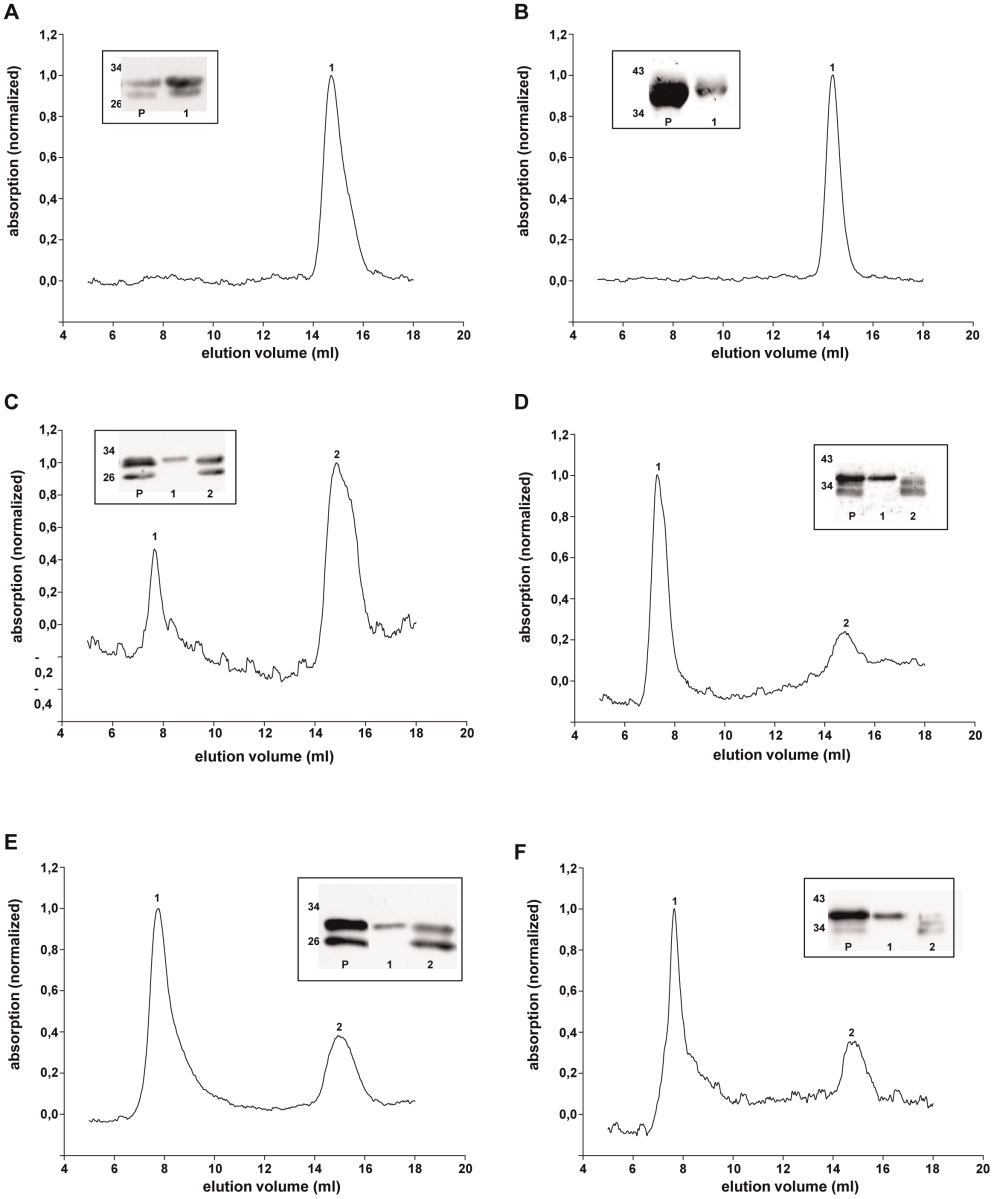
Gel filtration analysis of S-HO isoforms after purification. (A) S-HO-1ΔC266 (B) S-HO-2ΔC289 (C) S-HO-1 (D) S-HO-2 (E) S-HO-1-CPR co-purification (F) S-HO-2-CPR co-purification. The elution volume of peak 1 is nearly the void volume of the column and corresponds to sizes >2000 kDa. Peak 2 corresponds to a size in the range of 40 kDa. Western Blots of the purified samples (P) and the gel filtration-peaks are shown in the small boxes, detected with the HO-1 antibody (A+C+E) or HO-2 antibody (B+D+F), respectively.

Gel filtration analysis of purified preparations of the carboxy-terminally deleted variants of HO-1ΔC266 (Figure 5A) and HO-2ΔC289 (Figure 5B) revealed a single peak around 40 kDa likely representing monomeric HO. In contrast, analysis of the respective full length enzymes indicates a difference between HO-1 and HO-2: While there was only a small peak around 2000 kDa representing higher ordered oligomers for HO-1 (Figure 5C, peak 1), the shift from monomers to higher ordered oligomers was almost complete for full length HO-2 (Figure 5D). Co-expression of CPR did not influence the amount of monomeric HO-2 (Figure 5F), but substantially reduced the amount of monomeric HO-1 and increased the peak representing higher ordered HO-1 complexes (Figure 5E). As expected gel filtration analysis of carboxy-terminally deleted HO-variants did not change in the absence or presence of CPR (data not shown).

### HO-CPR interaction revealed by FRET analysis

**Figure 6:**
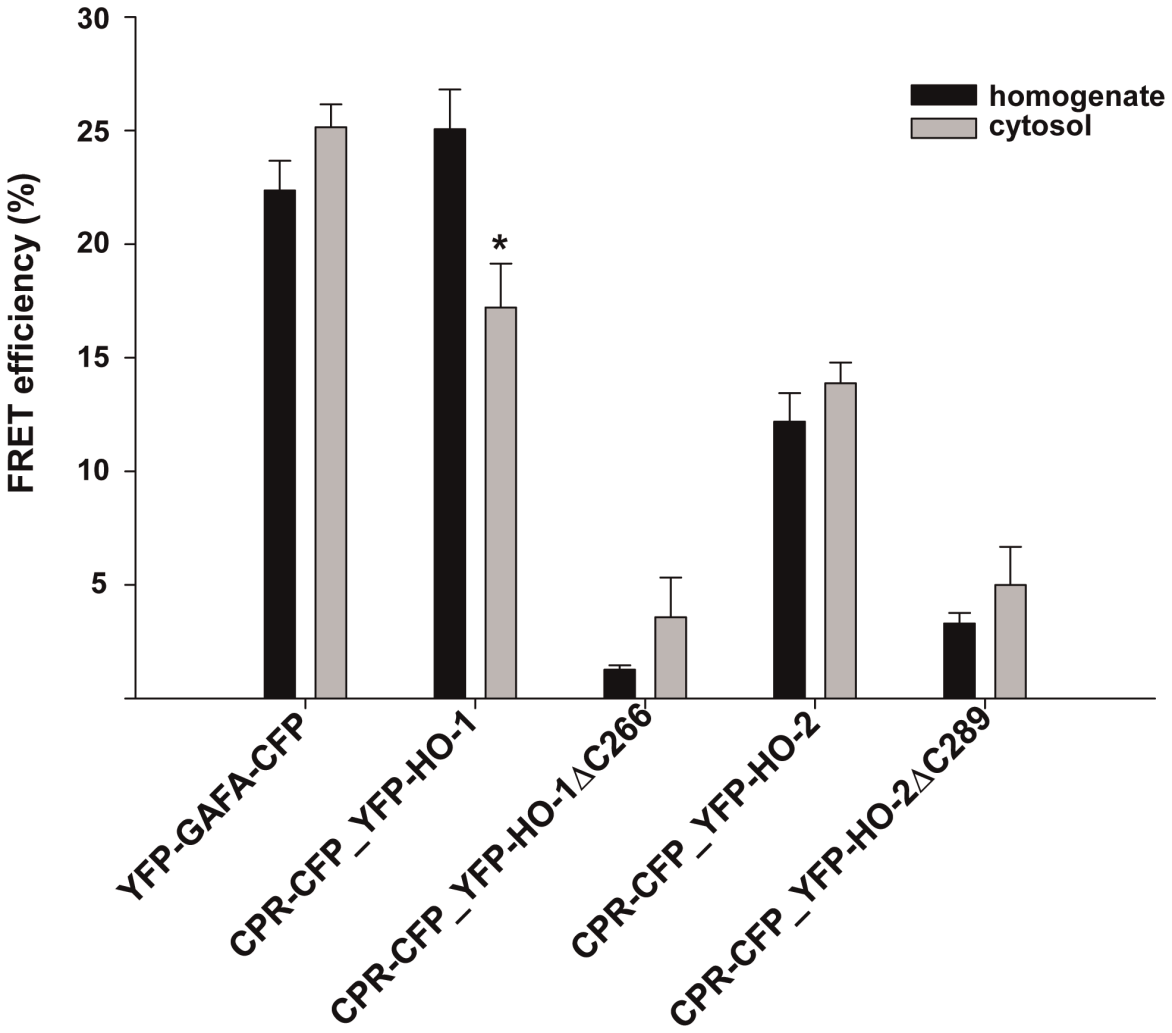
FRET measurements of HO isoforms and CPR in homogenate (black column) or cytosol (grey column). YFP-GAFA-CFP served as positive control for FRET-interactions. CPR was tagged with CFP and HO-variants with YFP. Data are shown as means ± SEM (n = 3, p<0.05).

As shown in Figure 1A and B, HO-activity of HO-1 but none of the other HO-forms differs in cytosol versus homogenate. In order to analyze the physical interaction of HO-variants with CPR in Sf9 cytosol and homogenate, we compared the respective Sf9 cell fractions by using FRET measurements. CPR was tagged with CFP as donor and the HO variants with YFP as acceptor. Our results indicate that HO-1 but none of the other HO-forms shows a significantly lower FRET efficiency in cytosol versus homogenate (Figure 6). This supports the idea that loss of the membrane anchor of HO-1 upon preparation of cytosol from homogenate leads to a decreased capacity of HO-1 to interact with CPR. This is supported by the expected finding that FRET efficiencies were highest for full length HO-1 and HO-2 and lowest for the respective carboxy-terminal deletion mutants. The FRET efficiency of HO-1 was in the same range as the positive control YFP-GAFA-CFP. In this construct previously characterized by our group both FRET-donor and FRET-acceptor are covalently linked by a protein [25].

### HO-CPR interaction in intact cells revealed by FLIM-FRET analysis

**Figure 7:**
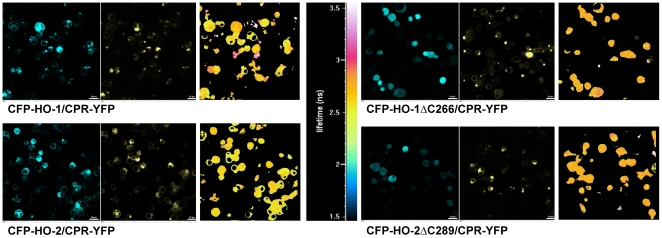
FLIM measurements of CFP-HOs with CPR-YFP in HEK293 cells. Pictures of the HO-CPR co-transfections were made in the CFP channel (left panels) and YFP channel (middle panels). The right panels show the color coded lifetime of the donor CFP. The corresponding color scale is shown in the center of the figure. The white bars correspond to 20 µm.

To measure FRET in intact cells we used FLIM (Figure 7). In the presence of co-transfected YFP-labeled CPR, full length HO-1 and HO-2 displayed FRET efficiencies of 10% and 7%, while the carboxy-terminal deletion mutants showed no significant FRET efficiency over baseline (0–1%) (Table 1). Thus, the membrane anchors of both HO-1 and HO-2 are important for efficient interaction with CPR in biochemical fractions as well as in intact cells.

**Table 1:** FRET efficiencies of HO-CPR co-transfections in HEK293 cells.

	FRET efficiency (%)	SEM (%)
**CFP-HO-1_CPR-YFP**	9.98	1.71
**CFP-HO-2_CPR-YFP**	6.86	1.19
**CFP-HO-1ΔC266_CPR-YFP**	1.17	0.67
**CFP-HO-2ΔC289_CPR-YFP**	−0.74	1.32

FRET efficiencies (%) with SEM were calculated after measuring the lifetimes of the samples in HEK 293 cells. CFP-HO-1 single transfection served as donor control. The mean calculation was carried out after three independent measurements.

### Translocation of HO-1 but not HO-2 under hypoxia

**Figure 8:**
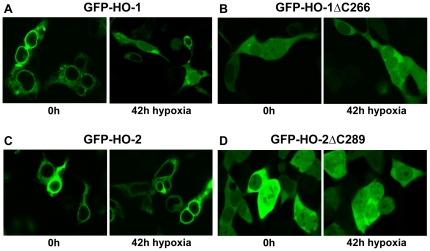
Confocal laser scanning analysis of GFP-tagged HO isoforms in HEK293 under hypoxia. The left panels show the cells at time 0 h and the right panels after incubation with 1% oxygen for 42 h. (A) GFP-HO-1; (B) GFP-HO1ΔC266; (C) GFP-HO-2; (D) GFP-HO-2ΔC289.

The carboxy-terminal part of HO-1 is important for subcellular localization to the endoplasmic reticulum [6], [10], [26]. Confocal microscopy of HEK293 cells transfected with amino-terminal GFP-fusion proteins revealed a granular perinuclear appearance for full length HO-1 (Figure 8A, left panel) and a homogenous cytosolic and nuclear fluorescence for GFP-HO-1ΔC266 (Figure 8B, left panel). An identical granular perinuclear appearance consistent with a localization to the endoplasmic reticulum was apparent for the GFP-fusion with full length HO-2 (Figure 8C, left panel). Again the carboxy-terminally deleted form, GFP-HO-2ΔC289, showed a homogenous cytosolic and nuclear fluorescence (Figure 8D, left panel).

Cell hypoxia leads to a translocation of HO-1 from the endoplasmic reticulum to the cytosol and the nucleus. There it can modulate DNA-binding activity of transcription factors including AP-1 independently of its catalytic activity [10], [27]. We could confirm the translocation of HO-1 induced by hypoxia (Figure 8A) and could show that it is specific for HO-1 (Figure 8C): 30.1% of the cells transfected with HO-1 translocate to the nucleus (Table 2) and show a more homogenous cytosolic fluorescence after exposure to hypoxia but only 0.43% of HO-2 transfected cells. At the same time our results with HO-2ΔC289 predict that tryptic cleavage of the membrane anchor of HO-2 would lead to translocation (Figure 8D) in 100% of the cells in the same range like HO-1 ΔC266 (99.04%-Figure 8B).

**Table 2:** Translocation rate of HO isoforms before and after incubation with hypoxia.

	0 h		42 h	
	Translocation (%)	SEM (%)	Translocation (%)	SEM (%)
HO-1	1.59	1.59	30.10	3.54
HO-2	0	0	0.43	0.43
HO-1 ΔC266	100	0	99.04	0.96
HO-2 ΔC289	100	0	100	0

The rate of cells showing a translocation to the nucleus before (0 h) and after incubation with hypoxia (42 h) were determined in comparison to total number of cells. The results consider three independent experiments and are shown as means (%)± SEM(%).

### CPR prevents hypoxic translocation of HO-1

**Figure 9:**
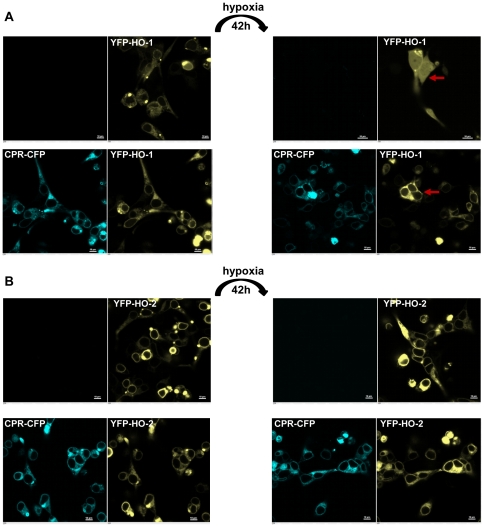
Influence of CPR on the hypoxic translocation of HO in HEK293 cells using confocal microscopy. HEK293 cells were transfected with YFP-HO-1 (A-row 1) and YFP-HO2 (B-row 1) or co-transfected with YFP-HO1 and CPR-CFP (A-row 2) or YFP-HO2 and CPR-CFP (B-row 2). Cells were analyzed under normoxic conditions using the CFP-channel (column 1) and YFP-channel (column 2) or after hypoxia in the CFP-channel (column 3) or YFP-channel (column 4). The red arrows in panel A point to typical cells where the translocation of HO-1 (row 1) and its prevention in the presence of CPR (row 2) are visible. The white bars correspond to 10 µm.

Because it has been shown that oligomerization is crucial for the stability of HO-1 [26], we hypothesized that the greater stability of HO-2 in Sf9 biochemical fractions and the greater resistance of HO-2 for tryptic cleavage of the membrane anchor under conditions of hypoxia may be due to its more complete oligomerization (see Figure 5D). Based on this idea, we tested whether co-expression of CPR which leads to higher ordered HO-1 complexes (see Figure 5E), may also modulate hypoxic translocation of HO-1 in intact cells. Figure 9A shows that co-expression of CPR prevents translocation of HO-1 under conditions of hypoxia (see red arrows). Figure 9B shows the analogous control experiment for HO-2.

## Discussion

In the present study we show for the first time that CPR prevents nuclear translocation of HO-1 under hypoxic conditions and promotes oligomerization of HO-1 into higher ordered complexes. In addition, we demonstrate that nuclear translocation of HO under hypoxic conditions occurs in an isoform specific manner for HO-1. Lastly our study is the first systematic analysis in a eukaryotic system that demonstrates the crucial role of the membrane anchor of HO-2 for localization at the endoplasmic reticulum, oligomerization and interaction with CPR.

It has been shown by Lin et al. 2007 that hypoxia leads to translocation of HO-1 to the nucleus which is associated with the truncation of its carboxy-terminus [10]. Our work on HO-1 in the current paper confirms these results. Nuclear carboxy-terminally truncated HO-1 was reported to be catalytically inactive [10]. This is based on an experiment using an enzyme lacking the last 23 amino acids [10]. At first this is surprising because carboxy-terminal deletion of 23 amino acids of HO-1 has previously been shown not to negatively affect HO activity [15] and we fully confirm this finding in the current study. However, as emphasized by our current work the catalytic inactivity of the carboxy-terminally deleted enzyme described in [10] can be explained by a lack of CPR in the nucleus.

Nuclear HO-1 was demonstrated to alter binding of transcription factors involved in oxidative stress independent on degradation of heme or production of carbon monoxide [10], [27]. Our work shows that CPR not only collaborates with HO-1 in its enzymatic function, but also influences the amount of nuclear HO-1 that alters gene expression in the nucleus. This is of particular relevance as CPR is a highly polymorphic gene [28], [29] and deficiency syndromes of CPR have been described in humans [30]. Mutations of CPR have recently been shown to differentially modulate HO-1 activity [31]. Our paper suggests that mutations in the CPR promoter [32] or CPR deficiency could increase nuclear HO-1 and lead to subsequent alterations in gene expression.

Based on FRET experiments, it has recently been demonstrated that oligomerization is crucial for stability and function of HO-1 in the endoplasmic reticulum. This oligomerization was shown to be strongly dependent on the presence of the intact carboxy-terminus of HO-1 [26]. We fully confirm these findings for HO-1 using alternate methods in the current paper. In addition, we extend the findings to HO-2 and show that oligomerization of full length HO-2 is more complete than oligomerization of full length HO-1 (see Figure 5B). Only when CPR is co-expressed, HO-1 reaches the oligomerization level of HO-2 (see Figure 5C). These results obtained by gel filtration are in line with a recent report that CPR has an influence on HO-1 oligomerization based on bioluminescence resonance energy transfer (BRET) [31]. Because of the nature of BRET it could only be stated that ‘CPR disrupted or otherwise influenced the conformation/configuration of the HO-1 multimers’ [31]. Our biochemical results using gel filtration support this notion and indicate that CPR increases HO-1 oligomerization.

It has been shown that oligomerization is crucial for the stability of HO-1 [26]. Our observation that co-expression of CPR increases HO-1 oligomerization and prevents tryptic cleavage of HO-1 during hypoxia is consistent with this finding. The higher degree of oligomerization of HO-2 reported in the current study, is a plausible explanation for the greater stability of HO-2 versus HO-1. However, it is also conceivable that the carboxy-terminus of HO-2 is inherently more stable or that it does not contain the HO-1 specific recognition sequence for a tryptic protease that is induced by hypoxia. This would also be possible, because the sequence of the carboxy-termini of HO-1 and HO-2 are not highly conserved (∼15%). It is not ruled out that there exists a signal in the intact cell that leads to tryptic cleavage of the HO-2 carboxy-terminus and nuclear translocation of HO-2. In fact, the occurrence of a carboxy-terminally deleted tryptic fragment of HO-2 has been described after overexpression in *E. coli* [33]. However, so far our experiments in eukaryotic cells indicate that HO-2 is quite stable and neither translocates in response to hypoxia nor in response to different hemin concentrations or hyperoxia (data not shown).

HO-2 has been suggested to function as an oxygen sensor for a calcium-sensitive potassium channel in glomus cells of the carotid and aortic bodies [34], [35], [36]: HO-2-derived CO maintains potassium channel activity in normoxia, while hypoxia leads to a reduction in CO formation, channel closure and depolarisation. While we demonstrate in the current paper that HO-2 does not translocate from the endoplasmic reticulum in response to hypoxia, HO-2 has recently been shown to be reversibly inactivated under hypoxic conditions by induction of the dithiol state of the carboxy-terminal heme regulatory motifs (see “Alignment S1”) and a subsequent increase of the K_d_ for heme well above endogenous heme concentrations [17], [37]. Thus, both HO-isoforms seem to have developed a mechanism of inactivation under conditions of hypoxia that fits their nature: The inducible HO-1 is inactivated by irreversible tryptic proteolysis and translocation away from the endoplasmic reticulum and away from CPR. The constitutive HO-2 is reversibly inactivated by the redox state of specific cysteines.

Besides the difference in reversibility there is another crucial difference between the inactivation mechanism of HO-1 and HO-2 in response to hypoxia. It has very recently been shown that there is an interdependence between HO activity and the activity of cytochrome P450 via competition for binding to CPR [38]. The translocation of HO-1 under conditions of hypoxia would increase the amount of CPR that can interact with cytochrome P450 [39], while the reversible inactivation of HO-2 under hypoxic conditions would not change its binding to CPR. As apparent in Figure 9, CPR has the capacity to inhibit HO-1 translocation under hypoxic conditions. This adds another layer of complexity to the equilibrium between HO-1, CPR and drug metabolizing cytochrome P450.

In sum, we demonstrate that HO-1 and HO-2 respond differently to hypoxia in the intact cell, that their oligomerization status is different and that CPR influences oligomerization and prevents hypoxic translocation of HO-1 but not of HO-2. From a pharmacological perspective, it is important to note that hypoxia may change the competitive binding equilibrium between HO-1, drug metabolizing cytochrome P450 and their common partner CPR [38]. Thus hypoxia may not only have a direct inhibitory effect on heme metabolism but also a possible indirect activating effect on drug metabolism.

## Supporting Information

Alignment S1 **Amino acid alignment of human HO-1 and human HO-2.** The carboxy-termini marked in yellow are missing in the HO-1ΔC266 and HO-2ΔC289 mutants. Amino acids marked in green and blue correspond to the CPR binding sites (11–12). Heme regulatory motifs are marked in red. Conserved amino acids are marked with “*”, while “:” and “.” describe strong and weak conservations, respectively (alignment made with ClustalW2).
(TIF)
