# Supplementary figures and images for: Heme Oxygenase Isoforms Differ in Their Subcellular Trafficking during Hypoxia and Are Differentially Modulated by Cytochrome P450 Reductase
Source: PLoS One. 2012 Apr 24;7(4):e35483. doi: 10.1371/journal.pone.0035483 (PMC3335857; doi:10.1371/journal.pone.0035483)

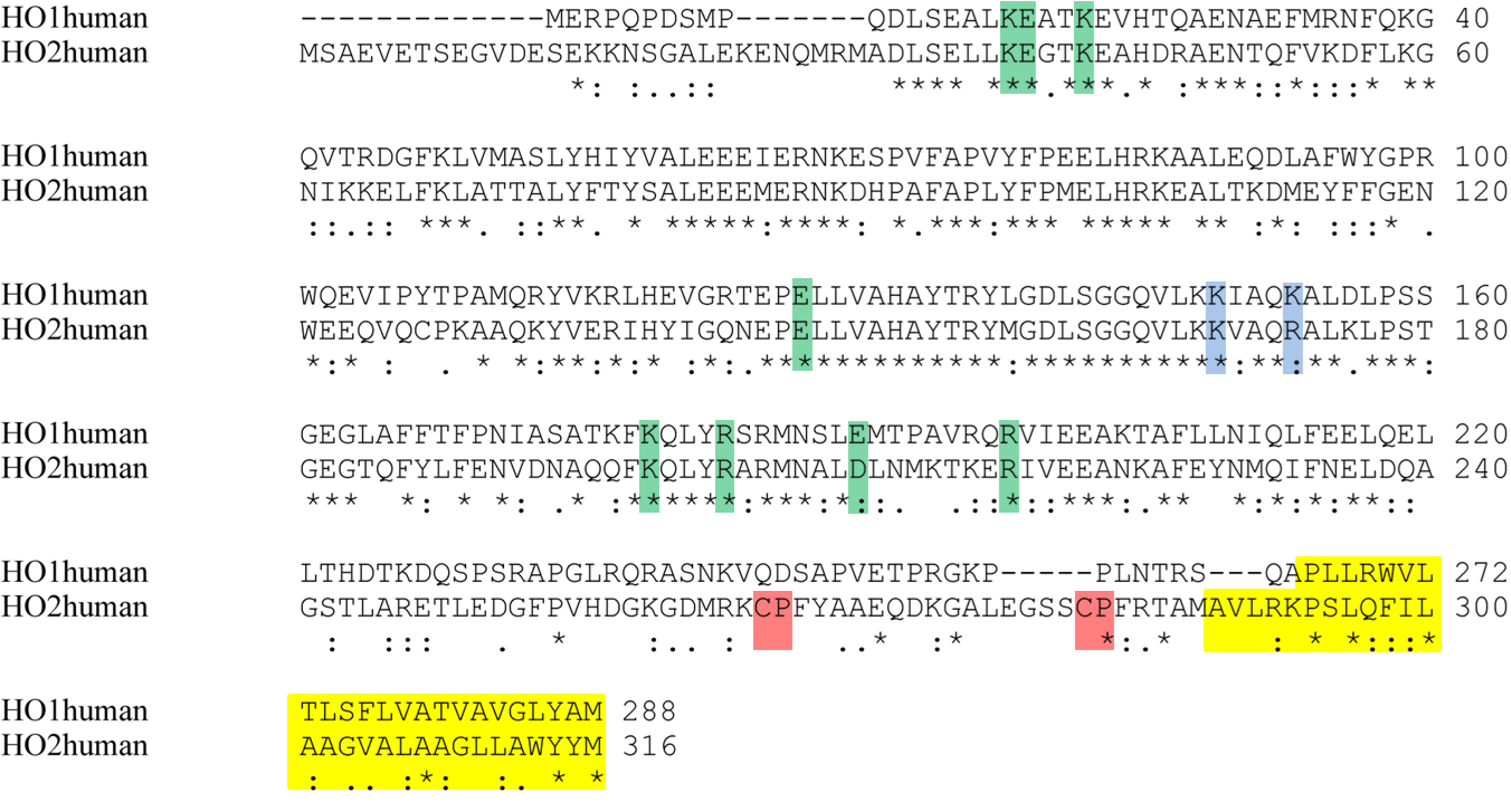

Supplement: Alignment S1 — Amino acid alignment of human HO-1 and human HO-2. The carboxy-termini marked in yellow are missing in the HO-1ΔC266 and HO-2ΔC289 mutants. Amino acids marked in green and blue correspond to the CPR binding sites (11–12). Heme regulatory motifs are marked in red. Conserved amino acids are marked with “*”, while “:” and “.” describe strong and weak conservations, respectively (alignment made with ClustalW2). (TIF) [file pone.0035483.s001.tif]
